# Nutritional, Antioxidant, and Functional Properties of Cameroonian Cowpea and Bambara Groundnut and Their Culinary Forms

**DOI:** 10.7759/cureus.74543

**Published:** 2024-11-26

**Authors:** Rosine Ornella Nameni, David Todem, Cerile Ypolyte Woumbo, Ronald Bidingha A. Goufani, Florian Amel Tekou, Ronice Zokou, Dieudonne Kuate, Jean-De-Dieu Tamokou

**Affiliations:** 1 Department of Biochemistry, University of Dschang, Dschang, CMR; 2 Department of Epidemiology and Biostatistics, Michigan State University, East Lansing, USA; 3 Department of Animal Biology, University of Yaounde I, Yaounde, CMR

**Keywords:** antioxidant, cowpea, culinary forms, functional property, nutritional value, voandzou

## Abstract

Background

Voandzou and cowpea are two legumes commonly used in African dishes as alternative sources of proteins. The present study aimed to evaluate the effects of steaming and frying on the nutritional and functional properties of Cameroonian cowpea (*Vigna unguiculata*) and Bambara groundnut (*Vigna subterranea*).

Methodology

The nutritional values, as well as the antioxidant, alpha-amylase, and alpha-glucosidase inhibitory activities of "koki" and "beignet koki," which are the traditional steamed dish and fritters made from cowpea and Bambara groundnut, respectively, were assessed.

Results

All the legumes displayed high levels of lipids, proteins, and carbohydrates. It was observed that both steaming and frying increased the lipid content and the energy values of cowpea and Bambara groundnut while simultaneously reducing the protein and carbohydrate contents. Both raw and processed legumes contained varying amounts of the minerals examined, with the treatments having inconsistent effects on them. The total phenolic content (TPC) and total flavonoid content (TFC) were significant in both legumes, with cowpea exhibiting the highest levels (TPC of 676.46 ± 16.87 µg gallic acid equivalents (GAE) per gram of extract and TFC of 219.27 ± 4.62 mg quercetin equivalents (QE) per gram of raw brown and black-eyed cowpeas, respectively). The antioxidant capacities, measured using different methods, were negatively impacted by both steaming and frying due to the destruction of their bioactive compounds. The alpha-amylase and alpha-glucosidase inhibitory capacities of both legumes experienced similar reductions after culinary treatments.

Conclusions

Cowpea and Bambara groundnuts are valuable sources of proteins, energy, and antinutrients. They are sufficiently rich in phenolic compounds with antioxidant and inhibitory effects on alpha-amylase and alpha-glucosidase. Consequently, these legumes can be used as functional foods. Stifled cooking and frying reduce mineral content (except for sodium), bioactive compound levels, and antioxidant, alpha-amylase, and alpha-glucosidase inhibitory capacities of the studied legumes.

## Introduction

Bambara groundnut and cowpea are two legumes commonly used in African dishes as alternative protein sources. A recent review [1] reported a global production of 8.9 million metric tons of cowpea alone, a starchy legume rich in protein and containing significant amounts of essential amino acids. Cowpeas have also been reported to have high iron and zinc content, making them an excellent option for vegetarians and people in countries where meat consumption is limited [2]. Notably, cowpeas contain nearly twice the amount of protein found in wheat [3]. These two legumes are cost-effective and can be used for both human and animal consumption [4]. However, despite the wide variety of dishes they can be used to prepare, they remain underutilized due to their strong bean flavor and significant antinutrient content, despite their high nutritional value. Oxalic acid, phytic acid, and tannins are the most commonly cited antinutrients, which can reduce the overall nutritional value of these foods [4]. Consequently, numerous studies have been conducted to investigate the effects of various processing methods on the nutritional value and antinutrient content of both Bambara groundnut and cowpea, in search of optimal methods that minimize the negative effects of these antinutrients on human health [1,5,6]. Interestingly, recent studies suggest that some of these antinutrients, in addition to the polyphenols and flavonoids found in these pulses, may play functional roles in humans, such as acting as antioxidants and exhibiting hypocholesterolemic and hypoglycemic effects [6]. Previous research has also demonstrated the antidiabetic potential of Bambara groundnut and cowpea [7], as well as their potential use in the development of functional foods and nutraceuticals due to their high levels of bioactive molecules such as polyphenols, flavonoids, and resistant starch [8]. In Cameroon and many other African countries, however, Bambara groundnut and cowpeas are primarily consumed in traditional dishes, such as "koki" [9], or fried after being ground and mixed with water to form a paste for producing fritters known locally as "beignet koki" [3], which are particularly popular among the Muslim population during fasting periods. Unfortunately, few studies have examined the effects of steaming and frying on the nutritional content, antinutrient levels, and biological activities of these two legumes. This study aims to address this knowledge gap by evaluating the effects of steaming and frying on the aforementioned properties of these two widely consumed legumes, as well as assessing their antidiabetic capacities as proposed in the literature.

## Materials and methods

Plant materials

Two varieties of *Vigna subterranea* (V1M and V5M) and *Vigna unguiculata* (LORI 1 and LORI 2) were obtained from the Agricultural Research Institute for Development (IRAD) in Garoua (Bénoué Department, North Cameroon) in November 2022. These legumes were then transported to the Research Unit of Biochemistry of Medicinal Plants, Food Sciences, and Nutrition (RUBPMAN) in the Department of Biochemistry at the University of Dschang, where they were cleaned and stored for future use.

Chemicals

We purchased alpha-amylase, alpha-glucosidase, Folin-Ciocalteu reagent, vanillin-hydrochloric acid, 1,1-diphenyl-2-picrylhydrazyl (DPPH), and 2,2'-azino-bis(3-ethylbenzothiazoline-6-sulfonic acid) (ABTS) from Sigma-Aldrich, Germany, and we prepared the reagents on the day of the experiments.

Preparation of *V. subterranea* and *V. unguiculata* fritters

The fritters of *V. subterranea* and *V. unguiculata* were prepared following a modified protocol [3]. The different legumes were soaked in distilled water for six hours at a ratio of 1/4 (w/v) and then manually pulped and ground using a Moulinex blender (Sokany, SK-444). Subsequently, 100 g of the crushed material was mixed with 70 mL of water, and 2 g of salt was added. The mixture was successively fried, by immersing 25 g of the mixture for six minutes in a pot (capacity of 5 L and 27 cm in diameter) placed on an electric heater (Alder, G-002), containing 2.5 L of palm oil at 150 °C. After frying, the fritters were cooled at room temperature for 20 minutes.

Preparation of dishes from *V. subterranea *and *V. unguiculata*

The modified protocol of Mbofung et al. [9] was used to prepare dishes from *V. subterranea* and *V. unguiculata*. The legumes were soaked in distilled water for six hours at a ratio of 1/4 (w/v). After manual pulping and grinding using a Moulinex blender (Sokany, SK-444), 200 g of the ground material was mixed with 150 mL of water, 7.6 mg of salt, and 120 mL of preheated palm oil. The entire mixture was stirred for 10 minutes, and then 90 mL of the resulting mixture was wrapped in banana leaves and boiled for 90 minutes in a pot (capacity of 5 L and 27 cm in diameter) placed on an electric heater (Alder, G-002), containing 1 L of boiling water, with banana leaves at the bottom to prevent water infiltration into the dishes. After cooking, the dishes were removed and cooled at room temperature for 30 minutes.

Determination of the nutritional content

Water, lipid, protein, fiber, ash, carbohydrate, and mineral (calcium, phosphorus, magnesium, iron, sodium, zinc, potassium) contents as well as the energy value were assessed following the standard methods [10].

Determination of the phytochemical composition

Phytate content was determined by maceration with hydrochloric acid followed by titration with FeCl₃•6H₂O [10]. Tannin content was determined using a vanillin-hydrochloric acid method [11].

For the determination of polyphenol and flavonoid contents, extracts were prepared by macerating 300 g of sample powder in 1 L of hexane for 72 hours, followed by filtration. The residues were further macerated in hot water for 24 hours, and the resulting filtrates were evaporated at 40 °C [12].

Polyphenol content was assessed using the Folin-Ciocalteu method. The absorbance of the extract was measured at 760 nm, and the polyphenol content was expressed as µg GAE per gram of dry extract, based on a calibration curve [13].

Flavonoid content was measured using an aluminum chloride colorimetric method [14]. The absorbance of the extract was read at 430 nm, and the flavonoid content was expressed as mg QE per gram of dry extract.

Antioxidant assays

The DPPH [15] and ABTS scavenging activities [16], as well as ferric-reducing antioxidant power (FRAP) [17], were used to evaluate the antioxidant properties. The amount of extract capable of reducing Fe^3+^ was expressed in µg of GAE per gram of dry extract (µg GAE/g dry extract), using the calibration curve with the equation y = 9.6901x + 0.0111.

Evaluation of functional properties

The functional properties were studied by assessing the ability of *V. subterranea* and *V. unguiculata* extracts to inhibit alpha-amylase and α-glucosidase activities.

The α-amylase inhibitory activity of *V. subterranea* and *V. unguiculata* was determined using a modified protocol [18], with acarbose as a standard. A total of 250 µL of extract (25, 50, and 100 µg/mL) was added to a test tube, and 250 µL of α-amylase solution (0.5 mg/mL) prepared in 2 mM phosphate buffer (pH 6.9) was added. The mixture was pre-incubated at 25 °C for 10 minutes, and then 250 µL of a 1% starch solution (in 2 mM phosphate buffer, pH 6.9) was added, followed by a further 10-minute incubation at 25 °C. The reaction was terminated by adding 500 µL of dinitrosalicylic acid (DNS) solution. The tubes were incubated in boiling water for five minutes and then cooled to room temperature. The reaction mixture was diluted with 5 mL of distilled water, and absorbance was measured at 540 nm using a spectrophotometer. A control was prepared under the same conditions but with distilled water replacing the extract. The α-amylase inhibitory activity was calculated as follows: Inhibition (%) = (Absorbance of control - Absorbance of the sample) x 100 / Absorbance of control.

The α-glucosidase inhibitory activity of *V. subterranea* and *V. unguiculata* extracts was assessed using the method described by Kazeem et al. [18] with modifications, employing α-glucosidase from *Bacillus stearothermophilus*. A 3 mM solution of p-nitrophenyl glucopyranoside (pNPG) was prepared in phosphate buffer (20 mM, pH 6.9). A 100 µL aliquot of α-glucosidase solution (1 unit/mL) was pre-incubated with 50 µL of extract at various concentrations (25, 50, and 100 µg/mL) for 10 minutes. Then, 50 µL of the substrate (pNPG) was added to initiate the reaction, and the mixture was incubated at 37 °C for 20 minutes. The reaction was stopped by adding 2 mL of Na_2_CO_3_ (100 mM). The activity of α-glucosidase was determined by measuring the yellow color of the product (p-nitrophenol) released from pNPG at 405 nm. Acarbose was used as the standard, and phosphate buffer as the control. The inhibition of α-glucosidase activity was calculated using the following formula: Inhibition (%) = (Absorbance of control - Absorbance of the sample) x 100 / Absorbance of control.

Statistical analysis

All experiments were conducted in triplicate, and the variables were expressed as means ± standard deviations. Graphs were generated using Excel 16 software (Microsoft® Corp., Redmond, WA). The results were statistically analyzed using SPSS software (version 22.0; IBM SPSS Statistics for Windows, Armonk, NY). One-factor ANOVA, followed by a Waller-Duncan post-hoc test, was used to compare the means of different groups at a significance level of 0.05.

## Results

Proximate composition and energetic values

The stifled brown Bambara groundnut (SBB) exhibited the highest lipid content (29.42 ± 0.06%), followed closely by the stifled brown cowpea (SBC) with 28.97 ± 0.03% (Table 1). In general, culinary treatments resulted in an increase in the lipid content of both Bambara groundnut and cowpea. The most pronounced effect was observed with stifled cooking, which led to the highest lipid content in both legumes (25.90 ± 0.01% to 29.42 ± 0.06%). This increase in lipid content can be attributed to the disruption of cellular membranes during cooking, thereby facilitating the release of lipids. The protein content of the legumes and their culinary forms is also presented in Table 1. It is evident that raw cowpea contains the highest protein levels, with the black-contoured cowpea (BLC) and brown cowpea (BC) showing values of 22.36 ± 0.01% and 21.09 ± 0.01%, respectively. Culinary treatments resulted in a decrease in the protein content in both legumes with frying having the greatest negative impact (14.37 ± 0.05% to 18.06 ± 0.01%). In terms of sugar content, raw legumes exhibited the highest levels, with white Bambara groundnut (WB) and black contour cowpea (BLC) displaying the greatest values of 60.97 ± 0.03% and 59.54 ± 0.02%, respectively. Culinary treatments resulted in a decrease in the sugar content of both Bambara groundnut and cowpea. The most pronounced effect was observed with stifled cooking, which led to the lowest sugar content in both legumes (25.92 ± 0.03% to 29.69 ± 0.05%). BLC (7.2 ± 0.02%) and BB (5.71 ± 0.02%) both showed the highest fiber content, while steamed BLC (SBLC) (25.50 ± 0.00%) and steamed brown contour cowpea (SBC) (24.90 ± 0.00%) had the highest water contents, resulting more probably to the water addition during preparation and also to water absorption during the cooking. Stifled BB (SBB) and steamed BC (SBC) exhibited the highest energy values, with 446.66 ± 0.02 and 437.21 ± 0.03 Kcal/100 g, respectively. Culinary treatments generally increased the energy values of all legume varieties, with stifled cooking having the most pronounced effect. It may be a result of the higher lipid and carbohydrate contents following moisture reduction.

**Table 1:** Proximate composition and energetic values of Bambara groundnut, cowpea, and their culinary forms. Data are shown as mean ± standard deviation (n = 3); in the same column, values with different superscripts (a-g) significantly differ at p < 0.05. BB: brown Bambara groundnut; WB: white Bambara groundnut; BC: brown contour cowpea; BLC: black contour cowpea; SBB: steamed brown Bambara groundnut; SWB: steamed white Bambara groundnut; SBC: steamed brown contour cowpea; SBLC: steamed black contour cowpea; FBB: fried brown Bambara groundnut; FWB: fried white Bambara groundnut; FBC: fried brown contour cowpea; FBLC: fried black contour cowpea

Samples	Lipid (%)	Protein (%)	Carbohydrate (%)	Ash (%)	Fiber (%)	Water (%)	Energy (Kcal/100 g)
BB	10.9 ± 0.02^a^	18.61 ± 0.02^b^	59.38 ± 0.03^c^	2.01 ± 0.01^a^	5.71 ± 0.02^d^	9.10 ± 0.00^b^	410.06 ± 0.02^a^
WB	6.6 ± 0.00^b^	20.11 ± 0.01^a^	60.97 ± 0.03^c^	3.01 ± 0.01^b^	4.76 ± 0.01^a^	9.30 ± 0.00^b^	383.72 ± 0.02^b^
BC	8.24 ± 0.00^c^	21.09 ± 0.01^a^	58.66 ± 0.04^a^	2.00 ± 0.00^a^	4.74 ± 0.01^a^	10.00 ± 0.00^b^	393.16 ± 0.02^c^
BLC	6.48 ± 0.01^b^	22.36 ± 0.01^c^	59.54 ± 0.02^a^	2.01 ± 0.01^a^	7.2 ± 0.02^b^	9.60 ± 0.00^b^	385.92 ± 0.01^b^
SBB	29.42 ± 0.06^d^	16.89 ± 0.04^d^	28.58 ± 0.02^e^	1.00 ± 0.00^c^	2.57 ± 0.02^c^	24.10 ± 0.00^a^	446.66 ± 0.02^d^
SWB	25.9 ± 0.01^e^	19.22 ± 0.03^b^	28.23 ± 0.03^e^	2.02 ± 0.02^a^	2.82 ± 0.02^c^	24.63 ± 0.03^a^	422.90 ± 0.04^e^
SBC	28.97 ± 0.03^d^	18.20 ± 0.03^b^	25.92 ± 0.03^b^	2.01 ± 0.01^a^	2.75 ± 0.01^c^	24.90 ± 0.00^a^	437.21 ± 0.03^d^
SBLC	26.32 ± 0.05^e^	17.47 ± 0.02^d^	29.69 ± 0.05^f^	1.01 ± 0.01^c^	5.19 ± 0.02^d^	25.50 ± 0.00^c^	425.52 ± 0.03^e^
FBB	20.88 ± 0.01^f^	14.37 ± 0.05^e^	41.34 ± 0.03^d^	1.01 ± 0.01^c^	1.61 ± 0.01^e^	22.40 ± 0.01^d^	410.76 ± 0.03^a^
FWB	16.58 ± 0.02^g^	16.14 ± 0.04^d^	43.97 ± 0.04^d^	2.00 ± 0.00^a^	2.02 ± 0.02^c^	21.30 ± 0.00^d^	389.66 ± 0.04^c^
FBC	19.12 ± 0.03^f^	18.06 ± 0.01^b^	37.70 ± 0.04^g^	1.01 ± 0.01^c^	1.02 ± 0.02^e^	24.10 ± 0.02^a^	395.12 ± 0.03^c^
FBLC	17.5 ± 0.01^g^	14.84 ± 0.04^e^	42.75 ± 0.05^d^	1.00 ± 0.00^c^	3.66 ± 0.01^f^	23.90 ± 0.00^a^	387.86 ± 0.02^c^

Mineral content

The BC displayed the highest magnesium content (184.03 ± 0.03 mg/100 g), while the BLC exhibited the highest levels of iron (8.28 ± 0.01 mg/100 g) and zinc (4.49 ± 0.01 mg/100 g) (Table 2). The WB had the highest calcium (58.28 ± 0.03 mg/100 g), phosphorus (657.01 ± 0.01 mg/100 g), and potassium (1012.24 ± 0.04 mg/100 g) contents, whereas fried black contour cowpea (FBLC) showed the highest sodium content (80.11 ± 0.02 mg/100 g). In general, culinary treatments resulted in a decrease in the mineral content of both Bambara groundnut and cowpea but increased their sodium content.

**Table 2:** Mineral composition of Bambara groundnut, cowpea, and their culinary forms. Data are shown as mean ± standard deviation (n = 3); in the same column, values with different superscripts (a-k) significantly differ at p < 0.05. BB: brown Bambara groundnut; WB: white Bambara groundnut; BC: brown contour cowpea; BLC: black contour cowpea; SBB: steamed brown Bambara groundnut; SWB: steamed white Bambara groundnut; SBC: steamed brown contour cowpea; SBLC: steamed black contour cowpea; FBB: fried brown Bambara groundnut; FWB: fried white Bambara groundnut; FBC: fried brown contour cowpea; FBLC: fried black contour cowpea

Samples	Magnesium (mg/100 g)	Calcium (mg/100 g)	Iron (mg/100 g)	Sodium (mg/100 g)	Phosphorus (mg/100 g)	Potassium (mg/100 g)	Zinc (mg/100 g)
BB	152.04 ± 0.04^a^	48.55 ± 0.05^b^	5.42 ± 0.04^c^	27.49 ± 0.01^a^	489.02 ± 0.02^b^	992.54 ± 0.04^c^	2.31 ± 0.03^d^
WB	184.01 ± 0.01^b^	63.14 ± 0.03^a^	6.47 ± 0.02^a^	27.26 ± 0.02^a^	657.01 ± 0.01^a^	1012.24 ± 0.04^a^	2.46 ± 0.01^d^
BC	185.03 ± 0.03^b^	38.86 ± 0.01^c^	7.77 ± 0.02^b^	34.86 ± 0.03^b^	470.21 ± 0.02^c^	933.43 ± 0.03^b^	3.57 ± 0.02^a^
BLC	184.03 ± 0.03^b^	58.28 ± 0.03^a^	8.28 ± 0.01^b^	34.83 ± 0.01^b^	501.56 ± 0.01^d^	992.55 ± 0.03^c^	4.49 ± 0.01^b^
SBB	112.02 ± 0.02^c^	24.27 ± 0.02^d^	3.03 ± 0.03^d^	40.48 ± 0.02^c^	388.72 ± 0.02^e^	401.44 ± 0.04^d^	0.97 ± 0.02^c^
SWB	144.04 ± 0.04^d^	34.01 ± 0.01^c^	4.03 ± 0.02^d^	42.82 ± 0.02^c^	411.3 ± 0.02^f^	618.26 ± 0.03^e^	1.02 ± 0.01^c^
SBC	128.01 ± 0.01^e^	14.54 ± 0.04^e^	4.58 ± 0.01^d^	51.26 ± 0.02^d^	194.4 ± 0.03^g^	578.79 ± 0.03^f^	1.88 ± 0.03^e^
SBLC	144.01 ± 0.01^d^	24.27 ± 0.02^d^	5.03 ± 0.03^c^	51.25 ± 0.01^d^	272.13 ± 0.03^h^	559.09 ± 0.02^g^	2.07 ± 0.02^d^
FBB	56.02 ± 0.02^f^	14.54 ± 0.04^e^	1.02 ± 0.02^e^	80.09 ± 0.01^e^	176.86 ± 0.01^g^	263.53 ± 0.03^h^	0.48 ± 0.02^f^
FWB	96.03 ± 0.03^g^	9.18 ± 0.03^f^	1.46 ± 0.04^e^	60.29 ± 0.04^f^	200.69 ± 0.01^i^	480.28 ± 0.01^i^	0.59 ± 0.01^f^
FBC	88.04 ± 0.04^h^	9.68 ± 0.03^f^	1.83 ± 0.03^e^	80.09 ± 0.01^e^	180.03 ± 0.03^g^	302.96 ± 0.01^j^	0.65 ± 0.01^f^
FBLC	112.02 ± 0.02^c^	9.55 ± 0.15^f^	1.96 ± 0.02^e^	80.11 ± 0.02^e^	220.74 ± 0.02^i^	362.05 ± 0.04^k^	0.93 ± 0.02^c^

Phytochemical composition

The total phenolic (TPC) and total flavonoid (TFC) contents of aqueous extracts from raw and cooked Bambara groundnut and cowpea are presented in Table 3, along with the levels of two antinutrients (phytate and tannin). The BC had the highest TPC (676.46 ± 16.87 µg GAE/g of extract), while the BLC exhibited the highest TFC (219.27 ± 4.62 mg/g QE). As shown, cowpeas generally had significantly higher TPC and TFC than Bambara groundnut. Culinary treatments resulted in a decrease in total phenolic, total flavonoid, phytate, and tannin contents in both legumes. Frying had the greatest impact on total phenolic, phytate, and tannin content, while flavonoid content had the greatest effect on stifled cooking.

**Table 3:** Phytochemical composition of Bambara groundnut, cowpea, and their culinary forms. Data are shown as mean ± standard deviation (n = 3); in the same column, values with different superscripts (a-i) significantly differ at p < 0.05 BB: brown Bambara groundnut; WB: white Bambara groundnut; BC: brown contour cowpea; BLC: black contour cowpea; SBB: steamed brown Bambara groundnut; SWB: steamed white Bambara groundnut; SBC: steamed brown contour cowpea; SBLC: steamed black contour cowpea; FBB: fried brown Bambara groundnut; FWB: fried white Bambara groundnut; FBC: fried brown contour cowpea; FBLC: fried black contour cowpea. TPC: total phenolic content; TFC: total flavonoid content; GAE: gallic acid equivalent; QE: quercetin equivalent; CE: catechin equivalent

Samples	TPC (µg GAE/g of extract)	TFC (mg/g QE)	Phytate (mg/100 g)	Tannins (mg CE/g)
BB	509.57 ± 17.58^a^	192.57 ± 9.94^b^	42.15 ± 0.02^c^	10.05 ± 0.01^d^
WB	551.80 ± 27.79^b^	184.55 ± 8.36^a^	36.44 ± 0.02^d^	10.57 ± 0.07^da^
BC	676.46 ± 16.87^c^	196.94 ± 7.97^b^	36.38 ± 0.02^d^	10.13 ± 0.00^d^
BLC	632.13 ± 6.53^d^	219.27 ± 4.62^c^	36.56 ± 0.05^d^	10.66 ± 0.01^a^
SBB	406.18 ± 13.71^e^	159.74 ± 9.74^d^	30.25 ± 0.01^a^	9.38 ± 0.01^b^
SWB	439.47 ± 15.35^f^	119.88 ± 1.34^f^	30.47 ± 0.01^a^	9.55 ± 0.05^bc^
SBC	605.23 ± 14.71^d^	184.47 ± 7.76^a^	24.12 ± 0.03^b^	9.26 ± 0.02^b^
SBLC	525.06 ± 14.94^a^	205.00 ± 11.17^c^	30.46 ± 0.05^a^	9.78 ± 0.06^c^
FBB	387.31 ± 37.45^g^	170.32 ± 9.51^e^	18.13 ± 0.01^e^	2.94 ± 0.00^e^
FWB	419.07 ± 13.03^f^	173.24 ± 7.90^e^	21.03 ± 0.01^b^	4.63 ± 0.09^f^
FBC	468.36 ± 5.68^h^	176.62 ± 4.025^e^	11.97 ± 0.00^f^	4.71 ± 0.04^f^
FBLC	333.73 ± 18.73^i^	209.54 ± 11.62^c^	17.99 ± 0.01^e^	5.85 ± 0.00^g^

Antioxidant capacity

The antioxidant capacities of Bambara groundnut and cowpea, as well as their culinary forms, are presented in Table 4. The BLC showed the best antioxidant performance across all methods: DPPH radical scavenging activity (IC_50_ of 33.19 ± 0.12 µg/mL), ABTS scavenging activity (IC_50_ of 51.54 ± 0.09 µg/mL), and ferric-reducing antioxidant power (FRAP) (552.15 ± 6.79 µg GAE/g of extract). However, FBB showed the lowest antioxidant performance across DPPH (IC50 of 110.9 ± 0.18 µg/mL) and ABTS (IC50 of 124.50 ± 0.21 µg/mL) methods, while the SBB showed the lowest ferric-reducing antioxidant power (352.68 ± 16.57 µg GAE/g of extract).

**Table 4:** Antioxidant capacities of Bambara groundnut, cowpea, and their culinary forms. Data are shown as mean ± standard deviation (n = 3); in the same column, values with different superscripts (a-h) significantly differ at p < 0.05. BB: brown Bambara groundnut; WB: white Bambara groundnut; BC: brown contour cowpea; BLC: black contour cowpea; SBB: steamed brown Bambara groundnut; SWB: steamed white Bambara groundnut; SBC: steamed brown contour cowpea; SBLC: steamed black contour cowpea; FBB: fried brown Bambara groundnut; FWB: fried white Bambara groundnut; FBC: fried brown contour cowpea; FBLC: fried black contour cowpea; GAE: gallic acid equivalent; IC_50_: concentration of the sample that inhibits free radical activity by 50%; DPPH: 1,1-diphenyl-2-picrylhydrazyl; ABTS: 2,2'-azino-bis(3-ethylbenzothiazoline-6-sulfonic acid); FRAP: ferric-reducing antioxidant power; GA: gallic acid;  / : not determined

Samples	DPPH (IC_50_ in µg/mL)	ABTS (IC_50_ in µg/mL)	FRAP (µg GAE/g of extract)
BB	49.76 ± 0.10^a^	57.46 ± 0.10^b^	434.74 ± 1.43^c^
WB	38.54 ± 0.10^b^	51.89 ± 0.10^a^	476.82 ± 8.52^b^
BC	37.66 ± 0.09^b^	53.72 ± 0.10^a^	527.16 ± 5.03^a^
BLC	33.19 ± 0.12^c^	51.54 ± 0.09^a^	552.15 ± 6.79^d^
SBB	50.96 ± 0.11^a^	63.94 ± 0.10^c^	352.68 ± 16.57^e^
SWB	47.10 ± 0.08^a^	57.46 ± 0.09^b^	361.68 ± 14.46^f^
SBC	42.56 ± 0.10^d^	66.12 ± 0.13^c^	366.63 ± 8.47^f^
SBLC	42.15 ± 1.53^d^	59.05 ± 0.10^b^	372.79 ± 14.69^g^
FBB	110.9 ± 0.18^e^	124.50 ± 0.21^d^	367.04 ± 18.54^g^
FWB	58.93 ± 0.10^f^	69.01 ± 0.09^ce^	409.81 ± 6.11^h^
FBC	101.4 ± 0.10^e^	116.50 ± 0.14^d^	373.19 ± 4.85^g^
FBLC	42.56 ± 0.10^d^	76.27 ± 0.14^e^	416.73 ± 15.43^h^
GA	21.27 ± 0.19^g^	41.91 ± 0.10^f^	/

Functional properties

No significant difference was found between the alpha-amylase and alpha-glucosidase inhibitory activities of raw cowpea and Bambara groundnut (Table 5). However, the BLC exhibited the highest *in vitro* alpha-amylase inhibitory capacity (IC_50_ of 30.59 ± 0.12 µg/mL). All culinary treatments diminished the alpha-amylase inhibitory activity of both cowpea and Bambara groundnut, with frying having the greatest negative impact with IC_50_ range from 35.27 ± 0.08 µg/mL to 48.93 ± 0.57 µg/mL. Prolonged heat exposure likely reduced total phenolic and total flavonoid contents, which are the phytochemicals responsible for the observed inhibitory activities. Conversely, the WB showed the highest *in vitro* alpha-glucosidase inhibitory activity (IC_50_ of 8.84 ± 0.27 µg/mL), which can still be related to its phytochemical content.

**Table 5:** Alpha-amylase and alpha-glucosidase inhibitory activities of Bambara groundnut, cowpea, and their culinary forms. Data are shown as mean ± standard deviation (n = 3); in the same column, values with different superscripts (a-f) significantly differ at p < 0.05. BB: brown Bambara groundnut; WB: white Bambara groundnut; BC: brown contour cowpea; BLC: black contour cowpea; SBB: steamed brown Bambara groundnut; SWB: steamed white Bambara groundnut; SBC: steamed brown contour cowpea; SBLC: steamed black contour cowpea; FBB: fried brown Bambara groundnut; FWB: fried white Bambara groundnut; FBC: fried brown contour cowpea. FBLC: fried black contour cowpea; ACA: acarbose; IC_50_: half maximal inhibitory concentration

Samples	Alpha-amylase Inhibition (IC_50_ in µg/mL)	Alpha-glucosidase Inhibition (IC_50_ in µg/mL)
BB	33.69 ± 0.41^bc^	9.61 ± 0.29^a^
WB	32.18 ± 0.04^b^	8.84 ± 0.27^a^
BC	31.98 ± 0.95^b^	9.23 ± 0.24^a^
BLC	30.59 ± 0.12^b^	8.87 ± 0.44^a^
SBB	39.95 ± 0.24^a^	14.24 ± 0.24^b^
SWB	34.15 ± 0.29^c^	11.97 ± 0.27^c^
SBC	46.09 ± 0.55^d^	10.17 ± 0.24^d^
SBLC	44.03 ± 0.51^d^	12.16 ± 0.25^c^
FBB	48.93 ± 0.57^e^	17.13 ± 0.20^e^
FWB	48.67 ± 0.58^e^	15.44 ± 0.23^b^
FBC	37.89 ± 0.10^a^	10.53 ± 0.43^d^
FBLC	35.27 ± 0.08^c^	9.95 ± 0.22^d^
ACA	21.71 ± 0.45^f^	8.69 ± 0.30^a^

## Discussion

The present study aims to assess the effects of stifled cooking and frying on the nutritional and functional properties of Cameroonian cowpea (*V. unguiculata*) and Bambara groundnut (*V. subterranea*). Interestingly, the raw BB demonstrated a lipid content exceeding that reported in a previous study [4] for the brown Bambara groundnut cultivated in Zimbabwe (7.09 g/100 g). The lipid content of raw and cooked cowpeas ranged from 6.48 to 28.97%, figures which surpass those documented [1]. The protein content of cowpeas in this study is consistent with previously reported findings in the literature [1,19]. Stifled cooking and frying led to a notable reduction in the protein content across all legume varieties. However, stifled cooking appeared to mitigate protein loss more effectively than boiling, as it preserved a higher protein content, as observed in a previous study [1]. The reduction in protein content is likely attributable to heat sensitivity, resulting in protein degradation during cooking or leaching into the cooking fluids [6]. This argument is further supported by the observation that frying, which involves higher temperatures, resulted in a greater reduction in protein content compared to stifled cooking. The protein content of raw Bambara groundnut in this study was lower than the 23.25 g/100 g previously reported [4] for Zimbabwean BB. The sugar content of WB surpasses the 56.42 g/100 g previously reported [4] for raw BB. The carbohydrate content of cowpeas was similar to that reported in the literature [1]. Culinary treatments led to a decrease in carbohydrate content for both Bambara groundnut and cowpea, likely due to the breakdown of carbohydrates under heat or their loss during cooking [1], as well as their involvement in the Maillard reaction during frying.

WB had the highest calcium, phosphorus, and potassium contents, whereas FBLC showed the highest sodium content. Similar iron and zinc levels in raw and cooked cowpeas have been reported [2]. Except for sodium, which significantly increased after frying, all other culinary treatments caused a decrease in mineral content, likely due to the diffusion of minerals into the cooking medium (water or oil) during the process [5].

The TPC of raw and processed Bambara groundnut was consistent with values previously reported [8] from a study of 44 Bambara groundnut varieties across Burkina Faso and Chad. All culinary treatments reduced the phytochemical content in both Bambara groundnut and cowpea, likely due to the destruction of these compounds by heat during cooking or frying [6]. A previous study revealed that phenolic compounds are primarily found in legume coats, which are removed during processing, explaining the observed reduction in total phenolic and flavonoid contents after culinary treatments [2]. Furthermore, culinary treatments significantly reduced the antinutrient content of all legumes, with some showing more than a twofold decrease after frying. Stifled cooking and frying, or heat treatments in general, help destroy antinutrients and reduce their levels. Similar findings were reported by Ndidi et al. [5] for tannin and phytate content in Nigerian Bambara groundnut, although their reported values were higher than those in this study.

The findings of the present study also demonstrated the strong antioxidant capacity of the BLC through its ability to scavenge the radicals DPPH and ABTS and its FRAP, which can be attributed to its high phenolic, flavonoid, and tannin content, which are well-known for their antioxidant properties [8]. In fact, phenolic compounds (tannins, phenols, and flavonoids) are known to be potential antioxidants due to their ability to scavenge free radicals and active oxygen species such as singlet oxygen, superoxide anion radicals, and hydroxyl radicals. Consequently, the presence of such compounds could be responsible for the antioxidant activity found in the tested legumes. Similar results were obtained by Oyeyinka et al. [4] for brown and red Bambara groundnut cultivars, who also noted that heat treatment significantly reduced the antioxidant capacity of the studied legumes. This finding is consistent with the present study, where a significant decrease in DPPH and ABTS scavenging capacities and ferric-reducing capacity was observed across all legume varieties and their culinary forms. Raw cowpeas generally exhibited higher antioxidant activity than Bambara groundnut, and all culinary treatments led to a reduction in these activities. As previously noted by Okafor et al. [19] and Oyeyinka et al. [4], heat likely decomposes larger antioxidant molecules, such as phenolic compounds and tannins. A prior study by Nyau et al. [20] also demonstrated the antioxidant capacity of Bambara groundnut, highlighting the superiority of brown Bambara groundnut over other varieties. However, unlike Nyau et al. [20] who reported much lower antioxidant activity in their DPPH scavenging and ferric-reducing assays compared to the present study, these differences may point to the influence of geographical and environmental conditions on the bioactive compound content of legumes and their biological functions.

The BLC exhibited the highest alpha-amylase inhibitory capacity *in vitro*. This result can likely be attributed to its higher phytochemical and fiber content, as the literature suggests that many alpha-amylase and alpha-glucosidase inhibitors belong to the flavonoid, tannin, and saponin families [18]. However, culinary treatments significantly reduced the alpha-amylase and alpha-glucosidase inhibitory capacity of all legumes, with the greatest decrease after frying. Stifled cooking and frying destroy bioactive compounds (flavonoids and tannins) and reduce their activities [5].

Limitations

The main limitation of the study is the narrow scope of culinary methods and the variability in results for broader applicability. Another study will determine alternative cooking methods or strategies to preserve bioactive compounds.

## Conclusions

This study aimed to assess the effects of stifled cooking and frying on the nutritional and functional properties of Cameroonian cowpea (*V. unguiculata*) and Bambara groundnut (*V. subterranea*). Cowpeas and Bambara groundnuts are valuable sources of proteins, energy, and antinutrients. They are sufficiently rich in phenolic compounds with antioxidant and inhibitory effects on alpha-amylase and alpha-glucosidase. Consequently, these legumes can be used as functional foods. Stifled cooking and frying reduce mineral content (except for sodium), bioactive compound levels, and antioxidant, alpha-amylase, and alpha-glucosidase inhibitory capacities of the studied legumes.
